# Sarcopenia index as a predictor of clinical outcomes among older adult patients with acute exacerbation of chronic obstructive pulmonary disease: a cross-sectional study

**DOI:** 10.1186/s12877-023-03784-7

**Published:** 2023-02-11

**Authors:** Xuanna Zhao, Ruoxin Su, Rongwei Hu, Yujuan Chen, Xiaoyong Xu, Yalian Yuan, Jinhong Zhang, Wenchao Zhang, Yu Yang, Min Chen, Dongming Li, Bin Wu, Dan Huang, Dong Wu

**Affiliations:** 1 Department of Respiratory and Critical Care Medicine, Affiliated Hospital of Guangdong Medical University, Zhanjiang, China; 2 Department of Geriatrics, Affiliated Hospital of Guangdong Medical University, Zhanjiang, China

**Keywords:** Sarcopenia index, Creatinine, Cystatin C, Elderly patients, Acute exacerbation of chronic obstructive pulmonary disease, Clinical outcomes

## Abstract

**Background:**

Sarcopenia is a geriatric syndrome with progressive loss of skeletal muscle mass and function and has a negative impact on clinical outcomes associated with chronic obstructive pulmonary disease (COPD). Recently, the sarcopenia index (SI) was developed as a surrogate marker of sarcopenia based upon the serum creatinine to cystatin C ratio. We aimed to assess the value of SI for predicting clinically important outcomes among elderly patients with acute exacerbation of COPD (AECOPD).

**Methods:**

This cross-sectional study included elderly patients with AECOPD in China from 2017 to 2021. Clinical data were collected from medical records, and serum creatinine and cystatin C were measured. Outcomes included respiratory failure, heart failure, severe pneumonia, invasive mechanical ventilation, and mortality. Binary logistic regression was used to analyze the association between SI and clinical outcomes.

**Results:**

A total of 306 patients (260 men, 46 women, age range 60–88 years) were enrolled in this study. Among the total patients, the incidence of respiratory failure and severe pneumonia was negatively associated with SI values. After adjusting for potential confounding factors, binary logistic regression analyses showed that a higher SI was still independently associated with a lower risk of respiratory failure (odds ratio [OR]: 0.27, 95% confidence interval [CI]: 0.13–0.56, *P* < 0.05). In subgroup analysis, the incidence of respiratory failure was negatively associated with SI values in groups with both frequent exacerbation and non-frequent exacerbation. After adjustment for potential confounders, binary logistic regression analyses showed that a higher SI was also independently associated with a lower risk of respiratory failure in both groups (OR: 0.19, 95% CI: 0.06–0.64 and OR: 0.31, 95% CI: 0.11–0.85). However, there were no significant differences in the correlations between SI and the risk of heart failure, invasive mechanical ventilation, and mortality in all groups.

**Conclusion:**

The SI based on serum creatinine and cystatin C can predict respiratory failure in patients with AECOPD and either frequent or infrequent exacerbations. This indicator provides a convenient tool for clinicians when managing patients with AECOPD in daily clinical practice.

**Supplementary Information:**

The online version contains supplementary material available at 10.1186/s12877-023-03784-7.

## Background

Chronic obstructive pulmonary disease (COPD) is one of the most common chronic diseases and an important health care problem in older adults [1]. On average, each patient with COPD generally experiences 0.5 to 3.5 episodes of acute exacerbation annually [2]. Previous studies have reported that exacerbation contributes to an accelerated decline in lung function, reduced health status and quality of life, and increased risk of death [3–5]. Therefore, the prevention of COPD exacerbation is an active area of research.

Sarcopenia is a syndrome characterized by a progressive decline in skeletal muscle mass, strength, and function in older people [6]. It is closely related to osteoporosis and frailty syndrome and can increases the risks for adverse health outcomes such as falls, physical disability, hospital admission, poor quality of life, and mortality risk [7, 8]. In patients with COPD, sarcopenia is a common comorbidity and its prevalence is estimated to range from 15 to 55% [9]. Studies have shown that sarcopenia has a negative impact on a range of COPD-related clinical outcomes, including exercise capacity, balance, quadriceps and handgrip strength, gait speed, and physical activity levels, which results in impaired physical capacity, reduced health-related quality of life, frequent hospital admissions, increased health care utilization, and even mortality [9–11]. In turn, COPD exacerbations can rapidly induce loss of muscle mass and function [12]. Together, these form a vicious circle that accelerates COPD progression. Consequently, early identification of sarcopenia in patients with COPD, especially acute exacerbation of COPD (AECOPD), has crucial importance in clinical practice.

Traditional screening tests recommended for sarcopenia have limited use in clinical practice owing to high costs, radiation exposure, and requirements for highly specialized personnel [13]. Recently, the sarcopenia index (SI; serum creatinine [Cr, mg/dL]/cystatin C [CysC, mg/L] × 100) has been recommended as a novel screening tool for sarcopenia [14, 15]. Because the SI can be conveniently obtained using serologic findings in hospitalized patients, the index has been received increasing research interest. Studies have found that SI can predict the risk of complications after hip fracture in older adults, the future incidence of major adverse cardiovascular events in patients with obstructive coronary artery disease, and can be a promising biomarker for depressive symptoms in men [13, 16, 17]. However, the value of the SI in patients with AECOPD has not been reported to date. Thus, we sought to evaluate the relationship between the SI and clinical outcomes in elderly patients with AECOPD.

## Methods

### Data source

The data used in this study were part of a national key research and development project on COPD in China; this was a national clinical registration study initiated in June 2017 that lasted for 3.5 years (Clinical Trials ID: NCT03187236).

### Study cohort

Study participants were inpatients from the Department of Respiratory and Critical Care Medicine and the Department of Geriatrics, Affiliated Hospital of Guangdong Medical University, China. All patients were over 60 years old, and diagnosed with AECOPD, following the 2017 Global Initiative for Chronic Obstructive Lung Disease (GOLD) [18]. That study was approved by the Ethics Committee of the Affiliated Hospital of Guangdong Medical University and was conducted in accordance with the Declaration of Helsinki. Participants were informed of the purpose of the study and signed a consent form. Between 2017 and 2021, 366 participants were enrolled in the study. Owing to the different clinical outcomes among patients with frequent exacerbation (participants who experienced two or more exacerbations within the past year) and without frequent exacerbation [19], we analyzed the association of SI and clinical outcomes separately according to the COPD phenotype. We excluded patients with incomplete data (*n* = 20), chronic kidney disease with serum Cr 2.0 mg/dL (*n* = 8) or acute kidney injury (increase in serum Cr levels to ≥ 1.5 times the baseline value that is known or presumed to have occurred within the prior 7 days) (*n* = 7), active cancer (*n* = 3), co-existing conditions such as musculoskeletal and neurological disorders (*n* = 2), and concomitant respiratory diseases other than COPD, such as asthma and bronchiectasis (*n* = 20). Thus, 306 participants were included in the final study sample.

### Clinical and laboratory measurements and spirometry

Demographic characteristics (age, sex, height, weight, body mass index (BMI), smoking history) and clinical characteristics, including comorbidities (diabetes, hypertension, coronary disease, and arrhythmia), COPD Assessment Test (CAT) score, modified Medical Research Council (mMRC) score, exacerbation history, arterial blood gas analysis, white blood cell (WBC), C-reactive protein (CRP), and albumin (ALB) were collected from the hospital database and the data management network (mzf.fwncpc.com).

Cr was measured via the picric acid method. Serum CysC was measured via latex enhanced immune turbidimetry; the assay details are as follows. Buffer-diluted serum samples were mixed with latex covalently bound antibodies to form stable antigen–antibody complexes, resulting in a certain degree of turbidity. By measuring the absorbance of the mixture and comparing it with the calibration solution under the same conditions, the concentration of CysC in the sample could be calculated. The SI was calculated using the following formula: serum Cr/CysC value × 100 [14]. According to the SI values, all participants were divided into two groups: low or high SI. The SI median was used as the cutoff value, with low SI defined as lower than the median and high SI defined as equal to or higher than the median. All measurements were conducted in our hospital’s clinical laboratory. We performed spirometry according to the guidelines for lung function tests formulated by the Chinese Thoracic Society. We measured percentage predicted forced expiratory volume in 1 s (FEV_1_% predicted) in all participants.

### Clinical outcome measures

Clinical outcomes, defined according to the International Classification of Disease Tenth Revision (ICD-10) (respiratory failure, heart failure, severe pneumonia, invasive mechanical ventilation, and mortality), were obtained from the hospital database.

### Statistical analysis

All statistical analyses were performed with IBM SPSS Statistics, Version 25.0, (IBM Corp., Armonk, NY, USA) with the statistical significance level set at *P* less than 0.05 (*P* < 0.05). Categorical data are reported as number (percentage). Continuous variables are presented as mean ± standard deviation (SD) for normally distributed data. The chi-square test was used for comparisons of categorical variables, and the independent *t*-test was used for the comparison of normally distributed continuous variables. Binary logistic regression analysis was used to analyze the association between SI and clinical outcomes. Two models were used in this regression analysis. Model 1 was unadjusted; model 2 was adjusted for confounder variables, including age, sex, smoking history, BMI, FEV_1_% predicted, WBC, CRP, ALB, GOLD grade (severity grading of COPD), CAT score, mMRC score, and comorbidities (diabetes, hypertension, coronary disease, arrhythmia). We adjusted for these variables, which we thought were related to the clinical outcomes.

## Results

### Demographic and clinical characteristics

In total, 306 patients with AECOPD were included: 260 (85.0%) male patients and 46 (15.0%) female patients (mean age: 71.29 SD: 7.09, range: 60–88 years). We divided all participants into two groups: those with non-frequent exacerbation and those with frequent exacerbation. In this study, 200 (65.4%) patients had non-frequent exacerbation and 106 (34.6%) had frequent exacerbation. Participants were further divided into two groups according to the median SI. Low SI was defined as SI < 91.75 for all participants, SI < 82.94 for participants in the frequent exacerbation group, and SI < 101.41 for participants in the non-frequent exacerbation group. Participants with SI equal to or above these medians were defined as having a high SI. We observed that the two groups differed significantly in BMI, FEV_1_% predicted, ALB level, GOLD grade, CAT score, and mMRC score among all participants. There were significant differences between the two SI groups for patients with non-frequent exacerbation in terms of BMI and ALB level; patients with frequent exacerbation differed in BMI between the low and high SI groups (Table 1).

**Table 1:** Baseline characteristics of participants according to the SI

Characteristics	Total (n = 306)		*P*	Frequent exacerbators (n = 106)		*P*	Non-frequent exacerbators (n = 200)		*P*
	low SI (SI < 91.75, n = 153)	high SI (SI ≥ 91.75, n = 153)		low SI (SI < 82.94, n = 53)	high SI (SI ≥ 82.94, n = 53)		low SI (SI < 101.41, n = 100)	high SI (SI ≥ 101.41, n = 100)	
Age (years), mean ± SD	**72.08 ± 7.12**	**70.5 ± 7.01**	**0.051**	**71.51 ± 7.6**	**70.87 ± 7.06**	**0.653**	**71.9 ± 6.85**	**70.8 ± 7.13**	**0.267**
BMI (kg/m2), mean ± SD	**18.65 ± 3.22**	**21.23 ± 3.45**	** < 0.001**	**18.08 ± 2.88**	**20.38 ± 4.14**	**0.001**	**19.36 ± 3.19**	**21.26 ± 3.43**	** < 0.001**
Male, n (%)	**126 (82.4)**	**134 (87.6)**	**0.201**	**43(81.1)**	**47(88.7)**	**0.278**	**81(81)**	**89(89.0)**	**0.113**
Current smoker, n (%)	**32 (20.9)**	**34 (22.2)**	**0.781**	**12(22.6)**	**9(17.0)**	**0.465**	**17(17)**	**28(28.0)**	**0.063**
FEV_1_% predicted, mean ± SD	**40.53 ± 17.17**	**45.01 ± 18.29**	**0.028**	**38.61 ± 17.66**	**36.47 ± 14.97**	**0.502**	**43.88 ± 17.19**	**47.2 ± 18.8**	**0.193**
WBC (× 10^9/L), mean ± SD	**8.28 ± 3.68**	**8.46 ± 3.21**	**0.636**	**9.08 ± 4.07**	**8.2 ± 2.99**	**0.206**	**8.06 ± 3.54**	**8.39 ± 3.22**	**0.494**
CRP (mg/L), mean ± SD	**23.21 ± 43.55**	**18.45 ± 36.83**	**0.302**	**21.65 ± 45.26**	**34.18 ± 56.14**	**0.209**	**16.82 ± 29.32**	**17.32 ± 36.02**	**0.914**
ALB (g/L), mean ± SD	**37.29 ± 4.05**	**39.53 ± 4.27**	** < 0.001**	**37.01 ± 3.52**	**38.26 ± 4.56**	**0.119**	**37.53 ± 4.34**	**40.10 ± 4.00**	** < 0.001**
CAT, mean ± SD	**19.45 ± 6.18**	**15.92 ± 6.3**	** < 0.001**	**20.62 ± 5.83**	**19.43 ± 7.13**	**0.350**	**17.01 ± 6.43**	**15.87 ± 5.77**	**0.188**
GOLD, n (%)			**0.007**			**0.918**			**0.135**
1–2	**34 (22.2)**	**58 (37.9)**		**9 (17.0)**	**10 (18.7)**	**0.800**	**30 (30)**	**43 (43.0)**	**0.056**
3–4	**119 (77.8)**	**95 (62.1)**		**43 (81.1)**	**44 (83.0)**	**0.800**	**70 (70)**	**57 (57.0)**	**0.056**
mMRC, n (%)			**0.018**			**0.423**			**0.616**
0–2	**82 (53.6)**	**96 (62.7)**		**25 (47.2)**	**29 (54.7)**		**60 (60.0)**	**64 (64.0)**	
3–4	**71 (46.4)**	**57(37.3)**		**28 (52.8)**	**24 (45.3)**		**40 (40)**	**36 (36.0)**	
Comorbidities, n (%)									
Diabetes	**13(8.5)**	**12(7.8)**	**0.835**	**6(11.3)**	**5(9.4)**	**0.750**	**6(6)**	**8(8.0)**	**0.579**
Hypertension	**42(27.5)**	**38(24.8)**	**0.603**	**13(24.5)**	**15(28.3)**	**0.659**	**23(23)**	**29(29.0)**	**0.333**
Coronary Disease	**14(9.2)**	**17(11.1)**	**0.570**	**4(7.5)**	**6(11.3)**	**0.506**	**10(10)**	**11(11.0)**	**0.818**
Arrhythmia	**24(15.7)**	**13(8.5)**	**0.054**	**7(13.2)**	**9(17.0)**	**0.587**	**12(12)**	**9(9.0)**	**0.489**

*SI* Sarcopenia index, *BMI* Body mass index, FEV_1_% predicted: Percentage predicted forced expiratory volume in 1 s, *WBC* White blood cells, CRP C-reactive protein, *ALB* Albumin, *GOLD* Global initiative for chronic Obstructive Lung Disease, *CAT* COPD Assessment Test, *mMRC* modified Medical Research Council, *SD* Standard deviation

GOLD grade 1–2: FEV_1_% predicted ≥ 50%; GOLD grade 3–4: FEV_1_% predicted < 50%

### Clinical outcomes

The clinical outcomes observed in this study included respiratory failure, heart failure, severe pneumonia, invasive ventilation, and mortality. In the total patients, the incidences of respiratory failure and severe pneumonia were significantly associated with low SI; the incidence of respiratory failure was also significantly associated with low SI in both the frequent exacerbation and non-frequent exacerbation groups. However, there were no significant differences in the incidence of heart failure, severe pneumonia, invasive mechanical ventilation, and mortality between the groups with low SI and high SI in the frequent exacerbation and non-frequent exacerbation groups (Table 2).

**Table 2:** Differences in the distribution of clinical outcomes between patients with low SI and high SI

Clinical outcomes	Total n = 306		*P*	Frequent exacerbators n = 106		*P*	Non-frequent exacerbators n = 200		*P*
	low SI (SI < 91.75, n = 153)	high SI (SI ≥ 91.75, n = 153)		low SI (SI < 82.94, n = 53)	high SI (SI ≥ 82.94 n = 53)		low SI (SI < 101.41, n = 100)	high SI (SI ≥ 101.41, n = 100)	
Respiratory failure, n (%)	**54 (35.3)**	**18 (11.8)**	** < 0.001**	**26 (49.1)**	**15 (28.3)**	**0.028**	**22 (22.0)**	**9 (9.0)**	**0.011**
Heart failure, n (%)	**33 (21.6)**	**20 (13.1)**	**0.050**	**13 (24.5)**	**14 (26.4)**	**0.824**	**17 (17.0)**	**9 (9.0)**	**0.093**
Severe pneumonia, n(%)	**17 (11.1)**	**7 (4.6)**	**0.033**	**9 (17.0)**	**6 (11.3)**	**0.403**	**5 (5.0)**	**4 (4.0)**	**0.733**
Invasive mechanical ventilation, n(%)	**14 (9.2)**	**6 (3.9)**	**0.064**	**8 (15.1)**	**4 (7.5)**	**0.220**	**4 (4.0)**	**4 (4.0)**	**1**
Mortality, n (%)	**2 (1.3)**	**0**	**0.156**	**1 (1.9)**	**0**	**0.315**	**1 (1.0)**	**0**	**0.316**

*SI* Sarcopenia index

In the total patients, model 1 showed that a higher SI was associated with the risk of respiratory failure and severe pneumonia. After adjustment for potential confounding factors, model 2 showed that a higher SI was only independently associated with a lower risk of respiratory failure (odds ratio [OR]: 0.27, 95% confidence interval [CI]: 0.13–0.56). In subgroup analysis, model 1 showed that a higher SI was associated with the risk of respiratory failure in both the frequent exacerbation and non-frequent exacerbation groups. After adjustment for potential confounders, model 2 showed that a higher SI was also independently associated with a lower risk of respiratory failure in both groups (OR: 0.19, 95% CI: 0.06–0.64 and OR: 0.31, 95% CI: 0.11–0.85). However, there were no significant differences in the correlations between SI and the risk of heart failure, severe pneumonia, invasive mechanical ventilation, and mortality for both groups (Table 3).

**Table 3:** Associations between the sarcopenia index and clinical outcomes

Clinical outcomes	Total		Frequent exacerbators		Non-frequent exacerbators	
	Model 1	Model 2	Model 1	Model 2	Model 1	Model 2
Respiratory failure						
low SI (ref)	**1**	**1**	**1**	**1**	**1**	**1**
high SI	0.24(0.14–0.44) **	0.27(0.13–0.56) **	0.41(0.18–0.92) **	0.19(0.06–0.64) **	0.35(0.15–0.81) *	0.31(0.11–0.85) *
Heart failure						
low SI (ref)	**1**	**1**	**1**	**1**	**1**	**1**
high SI	**0.55(0.3–1)**	**0.84(0.39–1.79)**	**1.1(0.46–2.65)**	**1.11(0.32–3.88)**	**0.48(0.2–1.14)**	**0.42(0.13–1.35)**
Severe pneumonia						
low SI (ref)	**1**	**1**	**1**	**1**	**1**	**1**
high SI	0.38(0.15–0.95) *	**0.44(0.15–1.31)**	**0.62(0.21–1.9)**	**0.14(0.02–1.13)**	**0.79(0.21–3.04)**	**0.53(0.08–3.6)**
Invasive mechanical ventilation, n(%)						
low SI (ref)	**1**	**1**	**1**	**1**	**1**	**1**
high SI	**0.41(0.15–1.08)**	**0.45(0.13–1.5)**	**0.46(0.13–1.63)**	**0.02(0–1.1)**	**1(0.24–4.11)**	**2.62(0.24–29.08)**
Mortality, n(%)						
low SI (ref)	**1**	**1**	**1**	**1**	**1**	**1**
high SI	**NA**	**NA**	**NA**	**NA**	**NA**	**NA**

*SI* Sarcopenia index,*re*: Reference

NA: unable to analyze owing to few outcome events

Model 1: non-adjusted model. Model 2: adjusted for age, sex, smoking history, BMI, FEV_1_% predicted, WBC, CRP, ALB, GOLD grade (severity grading of COPD), CAT score, mMRC score, and comorbidities (diabetes, hypertension, coronary disease, arrhythmia)

^*^ *P* < 0.05, **P < 0.01

## Discussion

In this cross-sectional study, we investigated a sample of 306 patients with AECOPD between the ages of 60 and 88 years to evaluate the role of SI in predicting clinical outcomes among elderly patients with AECOPD. We also reported stratified results according to the AECOPD phenotype.

Our results showed that, regardless of group (total, frequent exacerbation group, or non-frequent exacerbation group), the incidence of respiratory failure was negatively associated with SI values. After adjustment for potential confounding factors, binary logistic regression analyses showed that a higher SI was also independently associated with a lower risk of respiratory failure, suggesting that the SI is a stable indicator predicting the risk of respiratory failure among patients with AECOPD. The SI is a simple and low-cost indicator that has been recommended as a novel screening tool for sarcopenia [15]. Poor nutritional status can contribute to decreased muscle mass and strength; therefore, nutritional status plays an important role in the development of sarcopenia [20, 21]. Owing to limited caloric intake and increased energy expenditure, patients with COPD complicated by respiratory failure often have protein-calorie malnutrition [22]. When the patient is having an acute episode of COPD, skeletal muscle dysfunction can be caused by hypoxia and/or hypercapnia, malnutrition, a strong inflammatory response, and the use of corticosteroids [23]. Therefore, sarcopenia is highly prevalent among patients with AECOPD complicated by respiratory failure. Furthermore, studies have shown that sarcopenia can have an impact on diaphragm muscle fatigue and can reduce respiratory muscle strength, including maximum inspiratory muscle pressure and maximum expiratory pressure [24–26]. These findings suggest that sarcopenia can increase the risk of respiratory failure in patients with AECOPD. Hence, we believe that the SI may predict respiratory failure among patients with AECOPD. Respiratory failure is a common complication in these patients. In severe cases, patients may be agitated, combative, or delirious, or they may lose consciousness and fall into a coma, thereby endangering their lives [27, 28]. Previous studies have shown that respiratory failure can increase the frequency of admissions to the emergency department or intensive care unit and is associated with significant mortality among patients with AECOPD [29, 30]. Therefore, routinely assessing the SI in the management of patients with AECOPD would facilitate more accurate early intervention, resulting in a reduced respiratory failure-related burden of the disease.

A systematic review and meta-analysis showed that sarcopenia and heart failure often co-exist, and the prevalence estimates of sarcopenia vary between 10 and 69% in patients with heart failure [31]. Both sarcopenia and heart failure share common pathophysiological pathways involving muscle dysfunction, which include alterations in mitochondrial density and activity, fiber distribution, and oxidative stress [32]. However, previous studies haven't focused on particular type of heart failure [33]. In our study, we did not find an association between SI and heart failure among older patients with AECOPD. The possible reason is that heart failure in our study was mainly right heart failure caused by chronic pulmonary heart diseases. It is well known that strong respiratory muscles can generate effective coughing that clears the airways and prevents pneumonia; in turn, if respiratory muscles are weak, this results in an increased propensity for pneumonia development. Sarcopenia is reported as a risk factor for the development of pneumonia in elderly people; several reports have also revealed the association between pneumonia and sarcopenia [16, 34, 35]. However, in our study, we detected no correlation between SI and severe pneumonia in patients overall, the group with frequent exacerbation, or the group without frequent exacerbation. Other studies have reported that sarcopenia is associated with mechanical ventilation and mortality among patients in the intensive care unit [36, 37], but there is no correlation between the SI and invasive mechanical ventilation or mortality among patients with AECOPD. It may be that the incidence of severe pneumonia, invasive mechanical ventilation, and mortality in our study was very low. More in-depth research with a larger sample size is needed.

Although as far as we know, this was the first study to evaluate the role of the SI in predicting clinical outcomes among patients with AECOPD, our study has certain limitations. First, this study was carried out at a single institution and included a small sample size. Additionally, most participants were men, which would lead to sex bias. Second, we did not have information for certain confounders, such as education level, income status, or medication complexity; these factors may confound the relationship between the SI and clinical outcomes among patients with AECOPD. Third, we did not use BIA or DXA to assess actual residual muscle mass in our patients. Finally, the inpatients in our study are not representative of patients with AECOPD in the general population or other clinical settings. Our sample is only representative of elderly patients with AECOPD in the Department of Respiratory and Critical Care Medicine and the Department of Geriatrics.

Sarcopenia is a complex and highly disabling disease that crucially affect the physical function and physical performance in elderly people with chronic disease, resulting in worsening disability, health-related quality of life, the need for assistance, detrimental implications for social and sanitary costs, and challenges in the therapeutic management [38]. Our study showed that the SI, based on serum Cr and CysC, can predict respiratory failure in elderly patients with AECOPD, suggesting that SI might be a simple, low-cost and stable indicator. Clinicians need to consider use SI as a critical indicator for the management of elderly patients with chronic diseases, including COPD.

## Supplementary Information

**Additional file 1.**

## Data Availability

The datasets used and/or analyzed during the current study available from the corresponding author on reasonable request.

## Abbreviations

COPD: Chronic obstructive pulmonary disease
AECOPD: Acute exacerbation of COPD
SI: Sarcopenia index
Cr: Creatinine
CysC: Cystatin C
GOLD: Global Initiative for Chronic Obstructive Lung Disease
BMI: Body mass index
CAT: COPD Assessment Test
mMRC: Modified Medical Research Council
WBC: White blood cell
CRP: C-reactive protein
ALB: Albumin
FEV_1_% predicted: Percentage predicted forced expiratory volume in 1 s
ICD-10: International Classification of Diseases Tenth Revision
SD: Standard deviation
OR: Odds ratio
CI: Confidence interval
BIA: Bioelectrical impedance analysis
DXA: Dual-energy x-ray absorptiometry
