# Supplementary material for: Sarcopenia index as a predictor of clinical outcomes among older adult patients with acute exacerbation of chronic obstructive pulmonary disease: a cross-sectional study
Source: BMC Geriatr. 2023 Feb 11;23:89. doi: 10.1186/s12877-023-03784-7 (PMC9921248; doi:10.1186/s12877-023-03784-7)
Supplement: Supplementary file 1 — Additional file 1. [file 12877_2023_3784_MOESM1_ESM.docx]

STROBE Statement—Checklist of items that should be included in reports of ***cross-sectional studies***

|  | Item No | Recommendation |
| --- | --- | --- |
| **Title and abstract** | 1 | 1. Indicate the study’s design with a commonly used term in the title or the abstract   **Title:** Sarcopenia index as a predictor of clinical outcomes among older adult patients with acute exacerbation of chronic obstructive pulmonary disease: a cross-sectional study |
|  |  | 1. Provide in the abstract an informative and balanced summary of what was done and what was found   **Abstract**  **Background:** Sarcopenia is a geriatric syndrome with progressive loss of skeletal muscle mass and function and has a negative impact on clinical outcomes associated with chronic obstructive pulmonary disease (COPD). Recently, the sarcopenia index (SI) was developed as a surrogate marker of sarcopenia based upon the serum creatinine to cystatin C ratio. We aimed to assess the value of SI for predicting clinically important outcomes among elderly patients with acute exacerbation of COPD (AECOPD).  **Methods:** This cross-sectional study included elderly patients with AECOPD in China from 2017 to 2021. Clinical data were collected from medical records, and serum creatinine and cystatin C were measured. Outcomes included respiratory failure, heart failure, severe pneumonia, invasive mechanical ventilation, and mortality. Binary logistic regression was used to analyze the association between SI and clinical outcomes.  **Results:** A total of 306 patients (260 men, 46 women, age range 60–88 years) were enrolled in this study. Among the total patients, the incidence of respiratory failure and severe pneumonia was negatively associated with SI values. After adjusting for potential confounding factors, binary logistic regression analyses showed that a higher SI was still independently associated with a lower risk of respiratory failure (odds ratio [OR]: 0.27, 95% confidence interval [CI]: 0.13–0.56, P < 0.05). In subgroup analysis, the incidence of respiratory failure was negatively associated with SI values in groups with both frequent exacerbation and non-frequent exacerbation. After adjustment for potential confounders, binary logistic regression analyses showed that a higher SI was also independently associated with a lower risk of respiratory failure in both groups (OR: 0.19, 95% CI: 0.06–0.64 and OR: 0.31, 95% CI: 0.11–0.85). However, there were no significant differences in the correlations between SI and the risk of heart failure, invasive mechanical ventilation, and mortality in all groups.  **Conclusion:** The SI based on serum creatinine and cystatin C can predict respiratory failure in patients with AECOPD and either frequent or infrequent exacerbations. This indicator provides a convenient tool for clinicians when managing patients with AECOPD in daily clinical practice. |
| Introduction | | |
| Background/rationale | 2 | Explain the scientific background and rationale for the investigation being reported  **Background**  Chronic obstructive pulmonary disease (COPD) is one of the most common chronic diseases and an important health care problem in older adults. On average, each patient with COPD generally experiences 0.5 to 3.5 episodes of acute exacerbation annually. Previous studies have reported that exacerbation contributes to an accelerated decline in lung function, reduced health status and quality of life, and increased risk of death. Therefore, the prevention of COPD exacerbation is an active area of research.  Sarcopenia is a syndrome characterized by a progressive decline in skeletal muscle mass, strength, and function in older people. It is closely related to osteoporosis and frailty syndrome and can increases the risks for adverse health outcomes such as falls, physical disability, hospital admission, poor quality of life, and mortality risk. In patients with COPD, sarcopenia is a common comorbidity and its prevalence is estimated to range from 15% to 55%. Studies have shown that sarcopenia has a negative impact on a range of COPD-related clinical outcomes, including exercise capacity, balance, quadriceps and handgrip strength, gait speed, and physical activity levels, which results in impaired physical capacity, reduced health-related quality of life, frequent hospital admissions, increased health care utilization, and even mortality. In turn, COPD exacerbations can rapidly induce loss of muscle mass and function. Together, these form a vicious circle that accelerates COPD progression. Consequently, early identification of sarcopenia in patients with COPD, especially acute exacerbation of COPD (AECOPD), has crucial importance in clinical practice.  Traditional screening tests recommended for sarcopenia have limited use in clinical practice owing to high costs, radiation exposure, and requirements for highly specialized personnel. Recently, the sarcopenia index (SI; serum creatinine [Cr, mg/dL]/cystatin C [CysC, mg/L] ×100) has been recommended as a novel screening tool for sarcopenia. Because the SI can be conveniently obtained using serologic findings in hospitalized patients, the index has been received increasing research interest. Studies have found that SI can predict the risk of complications after hip fracture in older adults，the future incidence of major adverse cardiovascular events in patients with obstructive coronary artery disease, and can be a promising biomarker for depressive symptoms in men. However, the value of the SI in patients with AECOPD has not been reported to date. |
| Objectives | 3 | State specific objectives, including any prespecified hypotheses  **Objective:** We sought to evaluate the relationship between the SI and clinical outcomes in elderly patients with AECOPD. |
| Methods | | |
| Study design | 4 | Present key elements of study design early in the paper  A cross-sectional study |
| Setting | 5 | Describe the setting, locations, and relevant dates, including periods of recruitment, exposure, follow-up, and data collection  The data used in this study were part of a national key research and development project on COPD in China; this was a national clinical registration study initiated in June 2017 that lasted for 3.5 years (Clinical Trials ID: NCT03187236). Study participants were inpatients from the Department of Respiratory and Critical Care Medicine and the Department of Geriatrics, Affiliated Hospital of Guangdong Medical University, China Between 2017 and 2021. That study was approved by the Ethics Committee of the Affiliated Hospital of Guangdong Medical University and was conducted in accordance with the Declaration of Helsinki. Participants were informed of the purpose of the study and signed a consent form.  Demographic characteristics (age, sex, height, weight, body mass index (BMI), smoking history) and clinical characteristics, including comorbidities (diabetes, hypertension, coronary disease, and arrhythmia), COPD Assessment Test (CAT) score, modified Medical Research Council (mMRC) score, exacerbation history, arterial blood gas analysis, white blood cell (WBC), C-reactive protein (CRP), and albumin (ALB) were collected from the hospital database and the data management network (mzf.fwncpc.com). |
| Participants | 6 | 1. Give the eligibility criteria, and the sources and methods of selection of participants   Study participants were inpatients from the Department of Respiratory and Critical Care Medicine and the Department of Geriatrics, Affiliated Hospital of Guangdong Medical University, China. All patients were over 60 years old, and diagnosed with AECOPD, following the 2017 Global Initiative for Chronic Obstructive Lung Disease (GOLD). That study was approved by the Ethics Committee of the Affiliated Hospital of Guangdong Medical University and was conducted in accordance with the Declaration of Helsinki. Participants were informed of the purpose of the study and signed a consent form. Between 2017 and 2021, 366 participants were enrolled in the study. We excluded patients with incomplete data (n=20), chronic kidney disease with serum Cr 2.0 mg/dL (n=8) or acute kidney injury (increase in serum Cr levels to ≥1.5 times the baseline value that is known or presumed to have occurred within the prior 7 days) (n=7), active cancer (n=3), co-existing conditions such as musculoskeletal and neurological disorders (n=2), and concomitant respiratory diseases other than COPD, such as asthma and bronchiectasis (n=20). Thus, 306 participants were included in the final study sample. |
| Variables | 7 | Clearly define all outcomes, exposures, predictors, potential confounders, and effect modifiers. Give diagnostic criteria, if applicable  Clinical outcomes, defined according to the International Classification of Disease Tenth Revision (ICD-10) (respiratory failure, heart failure, severe pneumonia, invasive mechanical ventilation, and mortality), were obtained from the hospital database. SI was used to predict clinically important outcomes among elderly patients with AECOPD. Potential confounders included age, sex, smoking history, BMI, FEV1% predicted, WBC, CRP, ALB, GOLD grade (severity grading of COPD), CAT score, mMRC score, and comorbidities (diabetes, hypertension, coronary disease, arrhythmia). |
| Data sources/ measurement | 8* | For each variable of interest, give sources of data and details of methods of assessment (measurement). Describe comparability of assessment methods if there is more than one group  Demographic characteristics (age, sex, height, weight, body mass index (BMI), smoking history) and clinical characteristics, including comorbidities (diabetes, hypertension, coronary disease, and arrhythmia), COPD Assessment Test (CAT) score, modified Medical Research Council (mMRC) score, exacerbation history, arterial blood gas analysis, white blood cell (WBC), C-reactive protein (CRP), and albumin (ALB) were collected from the hospital database and the data management network (mzf.fwncpc.com).  Cr was measured via the picric acid method. Serum CysC was measured via latex enhanced immune turbidimetry; the assay details are as follows. Buffer-diluted serum samples were mixed with latex covalently bound antibodies to form stable antigen–antibody complexes, resulting in a certain degree of turbidity. By measuring the absorbance of the mixture and comparing it with the calibration solution under the same conditions, the concentration of CysC in the sample could be calculated. The SI was calculated using the following formula: serum Cr/CysC value×100. All measurements were conducted in our hospital’s clinical laboratory. We performed spirometry according to the guidelines for lung function tests formulated by the Chinese Thoracic Society. We measured percentage predicted forced expiratory volume in 1 second (FEV_1_% predicted) in all participants.  Clinical outcomes, defined according to the International Classification of Disease Tenth Revision (ICD-10) (respiratory failure, heart failure, severe pneumonia, invasive mechanical ventilation, and mortality), were obtained from the hospital database. |
| Bias | 9 | Describe any efforts to address potential sources of bias  To reduce assessment bias, only one researcher (Dong Wu) with vast clinical expertise were responsible for making the diagnostics according to pre-specified criteria. The evaluator (Dan Huang) is a practicing respiratory physician with vast clinical expertise, who is well trained in the application of clinical scales. |
| Study size | 10 | Explain how the study size was arrived at  This is a cross-sectional study, the expected standard deviation (σ) of SI is 25, Assuming that α is 0.05, the two-sided $Z_{1-\alpha/2}$is 1.96, and the allowable error (δ) is 3. Based on the formula$n=\left( \frac{Z_{1-\alpha/2}\times\sigma}{\delta} \right)^{2}$, the sample size in 267. Considering a 10% dropout rate over the course of the study, the total sample size will be 296. |
| Quantitative variables | 11 | Explain how quantitative variables were handled in the analyses. If applicable, describe which groupings were chosen and why  Continuous variables are presented as mean ± standard deviation (SD) for normally distributed data, and the independent t-test was used for the comparison of normally distributed continuous variables. We analyzed the association of SI and clinical outcomes separately according to the COPD phenotype. According to the SI values, all participants were divided into two groups: low or high SI. The SI median was used as the cutoff value, with low SI defined as lower than the median and high SI defined as equal to or higher than the median. |
| Statistical methods | 12 | 1. Describe all statistical methods, including those used to control for confounding   The following statistical methods were used in this study: chi-square test, independent t-test, binary logistic regression analysis, and we adjusted for confounder variables, which we thought were related to the clinical outcomes. |
|  |  | 1. Describe any methods used to examine subgroups and interactions   All statistical analyses were performed with IBM SPSS Statistics, Version 25.0, (IBM Corp., Armonk, NY, USA) with the statistical significance level set at *P* less than .05 (*P* <.05). The chi-square test was used for comparisons of categorical variables, and the independent t-test was used for the comparison of normally distributed continuous variables. Binary logistic regression analysis was used to analyze the association between SI and clinical outcomes. Two models were used in this regression analysis. Model 1 was unadjusted; model 2 was adjusted for confounder variables, including age, sex, smoking history, BMI, FEV_1_% predicted, WBC, CRP, ALB, GOLD grade (severity grading of COPD), CAT score, mMRC score, and comorbidities (diabetes, hypertension, coronary disease, arrhythmia). We adjusted for these variables, which we thought were related to the clinical outcomes. |
|  |  | 1. Explain how missing data were addressed   The Case of missing data has been removed, a complete case analysis was performed. |
|  |  | (*d*) If applicable, describe analytical methods taking account of sampling strategy  We used convenience sampling. |
|  |  | 1. Describe any sensitivity analyses   We did not have any sensitivity analysis. |
| Results | | |
| Participants | 13* | 1. Report numbers of individuals at each stage of study—eg numbers potentially eligible, examined for eligibility, confirmed eligible, included in the study, completing follow-up, and analysed   Between 2017 and 2021, 366 participants were enrolled in the study. According to exclusion criteria, 60 patients were excluded. Thus, 306 participants were included in the final study sample. |
|  |  | 1. Give reasons for non-participation at each stage   We excluded patients with incomplete data (n = 20), chronic kidney disease with serum Cr 2.0 mg/dL (n = 8) or acute kidney injury (increase in serum Cr levels to ≥ 1.5 times the baseline value that is known or presumed to have occurred within the prior 7 days) (n = 7), active cancer (n = 3), co-existing conditions such as musculoskeletal and neurological disorders (n = 2), and concomitant respiratory diseases other than COPD, such as asthma and bronchiectasis (n = 20). |
|  |  | 1. Consider use of a flow diagram   INCLUDED EXCLUDED  Patients included in the Registry Study (n=366)  Patients with incomplete data  (n=20)  Patients with complete data (n=346)  Patients with chronic kidney disease with serum Cr 2.0 mg/dL (n=8), acute kidney injury(increase in serum Cr levels to ≥1.5 times the baseline value that is known or presumed to have occurred within the prior 7 days) (n=7), active cancer (n=3), co-existing conditions such as musculoskeletal and neurological disorders (n=2), concomitant respiratory diseases other than COPD, such as asthma and bronchiectasis (n=20)  Patients meeting baseline criteria for analysis (n=306) |
| Descriptive data | 14* | 1. Give characteristics of study participants (eg demographic, clinical, social) and information on exposures and potential confounders   In total, 306 patients with AECOPD were included: 260 (85.0%) male patients and 46 (15.0%) female patients (mean age: 71.29 SD: 7.09, range: 60–88 years). We divided all participants into two groups: those with non-frequent exacerbation and those with frequent exacerbation. In this study, 200 (65.4%) patients had non-frequent exacerbation and 106 (34.6%) had frequent exacerbation. Participants were further divided into two groups according to the median SI. Low SI was defined as SI <91.75 for all participants, SI <82.94 for participants in the frequent exacerbation group, and SI <101.41 for participants in the non-frequent exacerbation group. Participants with SI equal to or above these medians were defined as having a high SI. We observed that the two groups differed significantly in BMI, FEV_1_% predicted, ALB level, GOLD grade, CAT score, and mMRC score among all participants. There were significant differences between the two SI groups for patients with non-frequent exacerbation in terms of BMI and ALB level; patients with frequent exacerbation differed in BMI between the low and high SI groups (Table 1). |
|  |  | 1. Indicate number of participants with missing data for each variable of interest   The data included in the final analysis are complete data |
| Outcome data | 15* | Report numbers of outcome events or summary measures  The clinical outcomes observed in this study included respiratory failure, heart failure, severe pneumonia, invasive ventilation, and mortality. In the total patients, the incidences of respiratory failure and severe pneumonia were significantly associated with low SI; the incidence of respiratory failure was also significantly associated with low SI in both the frequent exacerbation and non-frequent exacerbation groups. However, there were no significant differences in the incidence of heart failure, severe pneumonia, invasive mechanical ventilation, and mortality between the groups with low SI and high SI in the frequent exacerbation and non-frequent exacerbation groups (Table 2). |
| Main results | 16 | 1. Give unadjusted estimates and, if applicable, confounder-adjusted estimates and their precision (eg, 95% confidence interval). Make clear which confounders were adjusted for and why they were included   In the total patients, model 1 showed that a higher SI was associated with the risk of respiratory failure and severe pneumonia. After adjustment for potential confounding factors, model 2 showed that a higher SI was only independently associated with a lower risk of respiratory failure (odds ratio [OR]: 0.27, 95% confidence interval [CI]: 0.13–0.56). In subgroup analysis, model 1 showed that a higher SI was associated with the risk of respiratory failure in both the frequent exacerbation and non-frequent exacerbation groups. After adjustment for potential confounders, model 2 showed that a higher SI was also independently associated with a lower risk of respiratory failure in both groups (OR: 0.19, 95% CI: 0.06–0.64 and OR: 0.31, 95% CI: 0.11–0.85). However, there were no significant differences in the correlations between SI and the risk of heart failure, severe pneumonia, invasive mechanical ventilation, and mortality for both groups (Table 3). |
|  |  | 1. Report category boundaries when continuous variables were categorized   The independent t-test was used for the comparison of normally distributed continuous variables (Table 1). |
|  |  | 1. If relevant, consider translating estimates of relative risk into absolute risk for a meaningful time period   We did not use relative risk. We used the odds ratio. |
| Other analyses | 17 | Report other analyses done—eg analyses of subgroups and interactions, and sensitivity analyses  We did not conduct other analyses in this study. |
| Discussion | | |
| Key results | 18 | Summarise key results with reference to study objectives  Our results showed that, regardless of group (total, frequent exacerbation group, or non-frequent exacerbation group), the incidence of respiratory failure was negatively associated with SI values. After adjustment for potential confounding factors, binary logistic regression analyses showed that a higher SI was also independently associated with a lower risk of respiratory failure, suggesting that the SI is a stable indicator predicting the risk of respiratory failure among patients with AECOPD. |
| Limitations | 19 | Discuss limitations of the study, taking into account sources of potential bias or imprecision. Discuss both direction and magnitude of any potential bias  First, this study was carried out at a single institution and included a small sample size. Additionally, most participants were men, which would lead to sex bias. Second, we did not have information for certain confounders, such as education level, income status, or medication complexity; these factors may confound the relationship between the SI and clinical outcomes among patients with AECOPD. Third, we did not use BIA or DXA to assess actual residual muscle mass in our patients. Finally, the inpatients in our study are not representative of patients with AECOPD in the general population or other clinical settings. Our sample is only representative of elderly patients with AECOPD in the Department of Respiratory and Critical Care Medicine and the Department of Geriatrics. |
| Interpretation | 20 | Give a cautious overall interpretation of results considering objectives, limitations, multiplicity of analyses, results from similar studies, and other relevant evidence  Our study showed that the SI, based on serum Cr and CysC, can predict respiratory failure in elderly patients with AECOPD who have either frequent or infrequent exacerbations. |
| Generalisability | 21 | Discuss the generalisability (external validity) of the study results  Clinicians should consider use of the SI in routine management of AECOPD not only to reduce the disabling sequelae of sarcopenia but also to optimize the comprehensive management of sarcopenia and COPD. |
| Other information | | |
| Funding | 22 | Give the source of funding and the role of the funders for the present study and, if applicable, for the original study on which the present article is based  This work was supported by the National Key Research and Development Program of China (grant number: 2016YFC1304404), the Discipline Construction Project of Guangdong Medical University (grant number: 4SG21231G), the Clinical Research Project of Affiliated Hospital of Guangdong Medical University (grant numbers: LCYT2017A003, LCYJ2020B008), the Young Innovative Talents Project of Universities in Guangdong Province (grant numbers: 2018KQNCX099), the Guangdong Province Medical Research Fund Mandatory Project (grant numbers: C2019016). |

*Give information separately for exposed and unexposed groups.

**Note:** An Explanation and Elaboration article discusses each checklist item and gives methodological background and published examples of transparent reporting. The STROBE checklist is best used in conjunction with this article (freely available on the Web sites of PLoS Medicine at http://www.plosmedicine.org/, Annals of Internal Medicine at http://www.annals.org/, and Epidemiology at http://www.epidem.com/). Information on the STROBE Initiative is available at www.strobe-statement.org.
